# Profiling Cognitive and Social Functioning in a Small Cohort with Malan Syndrome

**DOI:** 10.3390/children12020147

**Published:** 2025-01-27

**Authors:** Niccolò Butti, Cosimo Urgesi, Paolo Alfieri, Manuela Priolo, Rosario Montirosso

**Affiliations:** 1 Scientific Institute, IRCCS E. Medea, 0–3 Centre for the at-Risk Infant, 23842 Bosisio Parini, Lecco, Italy; 2 PhD Program in Neural and Cognitive Sciences, Department of Life Sciences, University of Trieste, 34127 Trieste, Italy; 3 Scientific Institute, IRCCS E. Medea, 33037 Pasian di Prato, Udine, Italy; 4 Laboratory of Cognitive Neuroscience, Department of Languages and Literatures, Communication, Education and Society, University of Udine, 33100 Udine, Italy; 5 Child & Adolescent Neuropsychiatry Unit, Bambino Gesù Children’s Hospital, IRCCS, 00165 Rome, Italy; 6 Medical and Molecular Genetics Unit, Azienda Ospedaliera di Rilievo Nazionale Cardarelli, 80131 Naples, Italy

**Keywords:** Malan syndrome, cognitive functioning, social functioning, neuropsychological profile, social perception, theory of mind, memory for faces, visual attention, comprehension of instructions, *NFIX* gene

## Abstract

**Background/Objectives**: Malan syndrome (MALNS) is an ultra-rare genetic disorder caused by aberrations in the *NFIX* gene, located at chromosome 19p13.2. Key features of MALNS include general overgrowth, a typical facial gestalt, muscle–skeletal abnormalities, speech difficulties and intellectual disability. Additionally, MALNS frequently presents with autism-like behaviour and social challenges. However, characterisation of the cognitive profile of MALNS, including social perception skills, is limited. **Methods**: Six children and adolescents with MALNS, whose clinical and emotional–behavioural features had been described in previous studies, were assessed by means of a single, co-normed neuropsychological battery covering multiple cognitive domains. **Results**: Consistent with their intellectual disability, performance was generally weak across all neuropsychological subtests. Nonetheless, memory for faces, visual attention and contextual (non-verbal) theory of mind emerged as relative strengths of the profile, both at group and individual levels. Conversely, tasks requiring verbal reasoning and language comprehension, such as comprehension of instructions and verbal theory of mind, represented weaknesses for all participants. **Conclusions**: These findings provide a further characterisation of cognitive and social functioning in MALNS, which can inform future research as well as clinical practice and rehabilitation

## 1. Introduction

Malan syndrome (MALNS; MIM#614753) is an ultra-rare disorder with an estimated prevalence of < 1/1,000,000 [1,2]. MALNS shares several clinical features with Sotos syndrome, as suggested by the previous definition of MALNS as Sotos-2 or Sotos-like syndrome. MALNS is due to haploinsufficiency of the nuclear factor I X gene (*NFIX*; MIM #164005), as a result of either heterozygous chromosomal microdeletions involving the 19p13.2 region or intragenic variants [3,4]. The main features include general overgrowth, a typical facial gestalt, muscle–skeletal abnormalities, speech difficulties and intellectual disability (ID). Although mild ID has been seldom documented [4], intellectual impairment usually ranges from moderate to severe [2].Visual impairments and brain abnormalities, such as Chiari I malformation, are other common features in MALNS [1,5].

As in other genetic disorders characterised by cognitive and social challenges such as Williams syndrome [6,7], Joubert syndrome [8] and Sotos syndrome [9], an accurate neuropsychological profiling of individuals with MALNS is an important goal to achieve a precise diagnosis and appropriate rehabilitation planning. In the last few years, some studies have attempted to characterise the cognitive phenotype in MALNS [10,11]. Mulder and colleagues (2020) shed light on specific features in the two allelic *NFIX*-related conditions, Marshall–Smith syndrome and MALNS by comparing seven individuals with MALNS and eight children with Marshall–Smith syndrome on behaviour, cognitive development and sensory processing [11]. In particular, individuals with MALNS showed greater difficulties in receptive than expressive language and in visuomotor integration than visual perception. Similarly, Alfieri and colleagues explored the cognitive, language and adaptive profiles in 15 individuals with MALNS, providing results similar to Mulder’s findings [10]. Still, the use of a non-homogenous assessment prevented direct comparisons between tests and, thus, a proper characterisation of the neuropsychological profile of MALNS.

The literature on MALNS also highlights comorbidities with anxiety, social attention problems, attention deficit and hyperactivity disorder (ADHD) and autism-like behaviour but minimal signs of autism spectrum disorder (ASD) [12]. A recent study using a remote, webcam-based tool reported weaker social attention in MALNS compared to the normative mean, but a high preference for social stimuli [13]. These mixed findings warrant further investigations of different components of social perception. Social perception skills encompass theory of mind (ToM) and facial affect recognition, and are defined as a set of cognitive abilities that enables an understanding of others’ emotions and mental states [14,15]. Deficits of social perception have been consistently reported in idiopathic ASD as well as in developmental disabilities that present comorbidities with ASD [16,17,18]. Interestingly, a recent parent-report, survey-based study documented a relatively low number of problems in social communication and interaction, with the exception of perspective taking [19]. This ability, directly involved in ToM tasks, was found to be particularly impaired across several neurodevelopmental genetic syndromes associated with ID and speech impairments. A direct assessment of social perception skills in individuals with MALNS may provide new insights into the social challenges and autistic features reported in this clinical population, and may shed light on possible dissociations between different components of social perception.

The current study aimed to explore the neuropsychological profile, including social perception skills, of a small cohort of children and adolescents with MALNS. All participants had previously undergone assessments of general cognitive functioning (i.e., IQ), adaptive behaviour, language, visuomotor integration and presence of emotional–behavioural and psychopathological problems, with results published in two recent studies [10,12]. In the current study, participants were evaluated by means of a single, co-normed neuropsychological battery, which also included subtests assessing social perception. This approach allowed for a more detailed description of cognitive and social functioning in MALNS, without requiring separate control groups.

## 2. Materials and Methods

### 2.1. Participants and Procedure

Families associated with Associazione Sindrome di Sotos Italia (ASSI) Gulliver, which is the Italian patient advocacy group devoted to both Sotos syndrome and MALNS, were informed about the opportunity to participate in the study. Interested families were then contacted by the researcher, who provided detailed information about the study’s aims and procedures of the study and arranged visits to the Scientific Institute, IRCCS E. Medea. Inclusion criteria were (i) genetic diagnosis of MALNS and (ii) age from 5 to 18 years. Genetic diagnosis was molecularly confirmed through clinical exome sequencing or comparative genomic hybridization/SNP array. These methods enabled the identification of intragenic *NFIX* pathogenic variants or *NFIX* microdeletions. While no additional information about the diagnostic process is available, as it was performed at other institutions, it is important to highlight that in Italy, informed consent is mandatory for genetic testing. Six children and adolescents aged 7–18 years, whose clinical and emotional–behavioural characteristics had been described in previous studies [10,12], were recruited. Two participants in the cohort were identified with microdeletions, while the remaining four had intragenic *NFIX* variants. Visual problems, such as polar cataracts and nystagmus, were prevalent across the cohort, aligning with the known clinical phenotype of MALNS. One participant (#1) presented with epilepsy, which was treated with valproic acid. Another participant (#4) exhibited EEG anomalies, which were not pharmacologically treated but were monitored through periodic EEG evaluations Regarding cognitive functioning, all participants presented with moderate-to-severe ID, except for one showing mild ID. In half of the sample, speech was absent or limited to a few words. Overall, adaptive behaviour scores were low across the cohort, particularly for those participants with severe ID [10]. Anxiety problems were documented in three participants, while participants #5 and #6 had received a diagnosis of ADHD [12]. Participant #3 was prescribed sertraline to manage symptoms of anxiety. Other common features included musculoskeletal problems such as scoliosis and pes planus, which are often associated with MALNS. It is important to note that the clinical and behavioural characteristics described here were reported in prior studies and not independently verified by the current study. All participants had either completed or were actively participating in various rehabilitative interventions, such as psychomotricity, physical therapy, speech therapy and occupational therapy. In terms of education, all participants were attending school, following differentiated and/or reduced programs adapted to their abilities. Each was supported by a special education teacher, as mandated by Italian laws, ensuring individualized attention and accommodations to promote learning and inclusion within the educational environment. Individual demographic and main clinical information is reported in Table 1.

### 2.2. Neuropsychological Assessment

The assessment took place during a short hospital stay (usually over two days), with the duration and number of sessions tailored to each child’s individual characteristics (e.g., age and behaviour). Participants were assessed with a set of subtests of the Italian version of the NEPSY-II battery [20,21]. These subtests were selected to measure multiple cognitive domains, including social perception, and to be administered to children of different ages and with cognitive and speech impairments. A brief description of the selected NEPSY-II subtests broken down into each neuropsychological domain is provided below.

- Attention and executive functions

Visual attention (VA): This is a paper–pencil cancellation task in which participants were asked to detect only figures matching specific target items. This subtest assessed selective visual attention and the ability to inhibit distractor information.

- Language

Comprehension of instructions (CI): Participants were required to indicate pictures on a sheet according to verbal commands given by the examiner. The instructions progressively increased in length and syntactic complexity. This subtest evaluated the ability to receive, process and execute oral instructions.

- Memory and learning

Memory for faces (MF): Participants were exposed to pictures of children’s faces and were later asked to identify these faces among other pictures. This subtest measured encoding, discrimination and recognition of facial features.

Memory for designs (MD): Participants were required to memorise coloured abstract drawings and their positions on a grid. They were then asked to select the previously seen drawings and place them correctly on a material grid. This subtest assessed spatial memory for novel visual material.

- Sensorimotor functioning

Manual motor sequences (MMS): Participants had to repeat a sequence of unimanual or bimanual gestures shown by the examiner. This subtest evaluated the ability to imitate rhythmic movement sequences.

- Social perception

ToM—verbal part (ToMA): Participants were presented with short stories or illustrations depicting social situations and answered questions requiring an understanding of another individual’s point of view to solve the task. This subtest measured the ability to understand mental functions and that others may have different thoughts and feelings.

ToM—contextual part (ToMB): Participants were asked to select the facial expression that appropriately represented the protagonist’s emotion in a given illustrated social context. This subtest assessed the ability to understand how emotion relates to social context.

Affect recognition (AR): This subtest assessed the ability to recognise affect and asked participants to discriminate facial affect expressions.

- Visuospatial processing

Block construction (BC): Participants used blocks to copy models or create three-dimensional representations of two-dimensional drawings. This subtest evaluated visuospatial construction skills.

Geometric puzzles (GP): Participants had to identify two geometrical shapes within a grid that matched figures outside the grid, which may have been rotated. This subtest assessed mental rotation, visuospatial analysis of abstract stimuli and attention to detail.

Given the anticipated floor performance, which could blur differences between subtests, raw scores were converted into scaled scores (mean = 10, SD = 3 and weak performance < 4) based on the mean and standard deviation from the Italian standardisation sample [21], without approximating values to the lowest extreme. This approach, used in prior studies on clinical populations with ID [8,22], provided scaled scores with a wider range (from −30 to 19) than that reported in normative standardisation tables (1–19), including negative values that reflected varying degrees of impaired performance. The use of a single, co-normed battery allowed subtest comparisons without requiring separate control groups, as a participant’s performance was compared to that of a large number of age-matched individuals with typical development included in the standardisation sample [23].

### 2.3. Data Handling and Statistical Analysis

For each NEPSY-II subtest, descriptive statistics (median and range) and the number of participants showing weak performance (scaled score < 4) were calculated. In order to further characterise the neuropsychological profile, exploratory analyses were performed using nonparametric tests, which were considered more appropriate given the small sample size and the expected non-normal distribution of participant performances across subtests. Specifically, a Friedman test was conducted, inserting the subtests’ scores as within-subject variables. Differences between specific subtests were then examined by means of Wilcoxon matched-pairs tests. Please note that the two parts of the ToM subtests were considered separately in order to explore potential dissociations between verbal and nonverbal components. All analyses were performed with Statistica software version 8.0 (Statsoft, Tulsa, OK, USA).

## 3. Results

Descriptive statistics and number of participants showing weaknesses in each subtest are reported in Table 2.

In line with the predominant moderate-to-severe ID observed in the sample, performance was generally low across all neuropsychological subtests. However, in VA, MF and ToMB, some participants did not show weaknesses, with even a participant (#6) exhibiting a performance above the mean in MF. Particularly low scores were observed in CI, MD, MMS and ToMA. AR and BC showed small variability, with all participants demonstrating weaknesses and their scores clustering roughly within an SD range of one. The Friedman test yielded a significant result (*χ*^2^_9_ = 33.27, *p* < 0.001), indicating the presence of distinct strengths and weaknesses within the cognitive profile. The follow-up Wilcoxon matched-pairs tests highlighted specific differences between subtests (Table 3).

**Figure 1:**
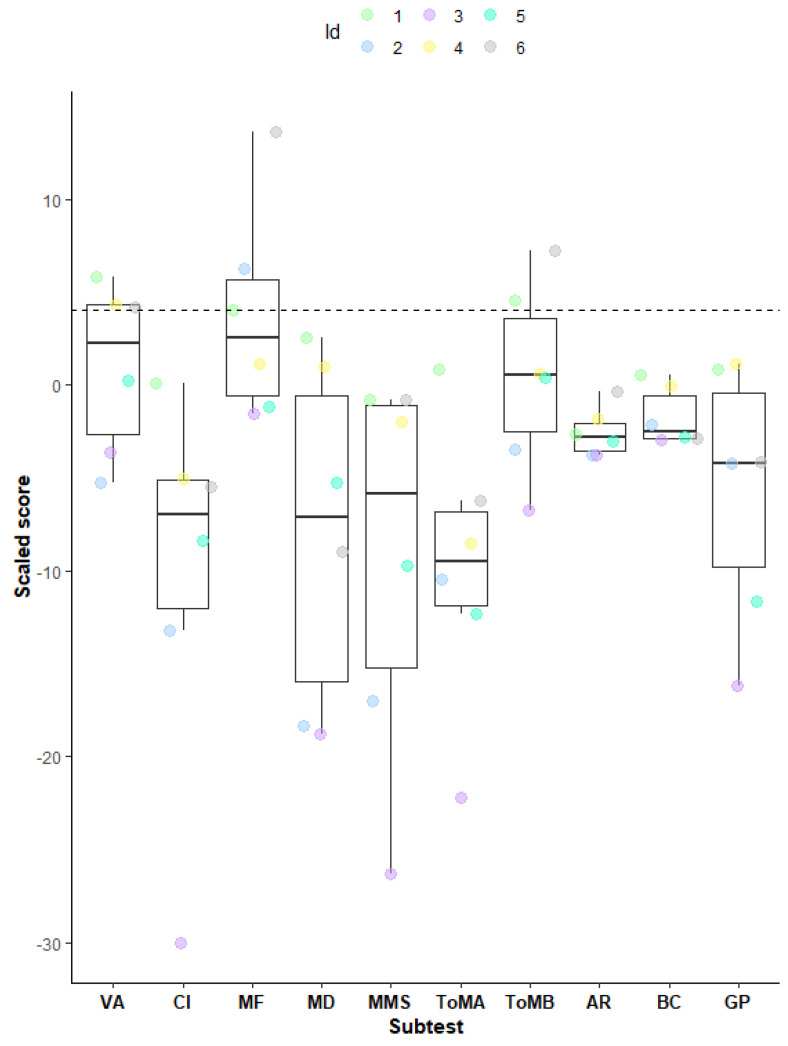
Boxplot of the subtest scores. The boxes represent the middle 50% of the data for each subtest. The upper and lower whiskers represent scores outside the middle 50% (i.e., the lowest and highest quartiles). The horizontal line within each box represents the median score. The dotted black line represents the threshold of weak performance (scaled score < 4). Coloured dots represent individual performance in each subtest. Legend: VA = Visual Attention; CI = Comprehension of Instructions; MF = Memory for Faces; MD = Memory for Designs; MMS = Manual Motor Sequences; ToMA = Verbal part of Theory of Mind; ToMB = Contextual part of Theory of Mind; AR = Affect Recognition; BC = Block Construction; GP = Geometric Puzzles.

Specifically, the best performance was observed in MF, with higher scores compared to all other subtests, except for VA and ToMB. These latter subtests also emerged as relative strengths, showing significantly better performance than CI, MD, MMS and GP, but not AR and BC. Conversely, CI and ToMA emerged as relative weaknesses, showing lower scaled scores than those obtained in MF, VA, ToMB, AR and BC. While low scores were observed across participants, MD, MMS and GP did not emerge as relative weaknesses or strengths in the profile. Participants with more severe ID (#2, 3, and 5) consistently showed lower scores across subtests, with participant #3 demonstrating particularly low performance in CI. Nonetheless, differences between subtests pointed to a specific neuropsychological profile, with relative strengths and weaknesses across the group (Figure 1).

## 4. Discussion

An assessment of neuropsychological phenotypes is widely recognized as crucial for a timely diagnosis and an early intervention; however, descriptions of the MALNS social and cognitive profile remain limited [10,11,12]. Such profiling is essential for ensuring comparability over time, particularly in the context of ultra-rare disorders as MALNS. Consistent with the presence of ID across the sample, performance was generally weak across neuropsychological subtests. However, visual, selective attention, memory for facial stimuli, and contextual and non-verbal ToM emerged as relative strengths, either when considering the group performance and at individual level. By contrast, language comprehension and verbal ToM skills were identified as relative weaknesses of all the profiled participants. The less affected performance observed in facial affect recognition and contextual ToM compared to the verbal part of the ToM subtest suggests greater difficulties in high-level, explicit socio-cognitive processes compared to nonverbal social perception skills. Despite being exploratory, these results add to previous seminal studies on cognitive and social functioning in MALNS [10,11,12], and they have important implications for assessment and rehabilitation.

The observed strengths in VA, MF and ToMB, and the relative weaknesses in CI and ToMA suggest a facilitation for tasks relying on visual processing over those requiring verbal reasoning and inference. Similar differences between visual and language skills have been previously reported for other genetic syndromes, such as Williams and Down syndromes [24]. However, rather than representing a specific dissociation, the differences between verbal and visual tasks reported here may depend on the different cognitive load involved in the adopted subtests. CI and ToMA require simultaneous processing and integration of visual and verbal input, while the VA, MF and ToMB subtests merely involve discrimination and retrieval of visual stimuli. In line with this hypothesis, which was previously proposed by Mulder and colleagues to explain similar patterns in WIPPSI-III subtests [11], a facilitation for visual stimuli was not found for MD and GP, which involve abstract stimuli and complex visuospatial processes such as spatial encoding and mental rotation.

Similarly, the varying cognitive demands of the social perception subtests may partially account for the greater difficulties in ToMA compared to ToMB and AR. Nonetheless, this difference also suggests that low-level, non-verbal processing of social information, such as facial affect recognition and a contextual, visual understanding of others’ mental states, may not be specifically impaired in MALNS. This interpretation is further supported by the strength observed at both the group and individual levels in MF, an ability often significantly impaired in ASD due to diminished interest in socially relevant stimuli [25]. By contrast, individuals with MALNS have been reported to exhibit a strong bias towards social information compared to abstract stimuli [13]. Overall, our findings help delineate the social phenotype of MALNS as distinct from that observed in idiopathic ASD, as described in other genetic syndromes presenting with autistic-like symptoms [26]. In line with this view, the behavioural profile of MALNS, described in a larger cohort that included the participants tested in the current study, reported frequent autism-like behaviours, attributed in part to language impairments and social anxiety observed in the study cohort, but minimal signs consistent with a formal diagnosis of ASD [12]. The discrepancy between contextual, non-verbal and verbal ToM skills observed in the present study also calls for further investigation of different levels of perspective taking, which has been indicated as particularly impaired in MALNS [19].

The adoption of a single, co-normed neuropsychological battery clarified findings from earlier studies that used tasks from different batteries and reported visuomotor and visuospatial difficulties [10,11,19]. In the current study, visuospatial and visuomotor subtests such as MD, MMS, BC and GP did not emerge as specific weaknesses of the profile. Indeed, visuomotor and visuospatial skills were very low compared to the normative mean, but greater difficulties were observed in individuals with more severe ID. These results suggest that, even though visuospatial and visuomotor skills may be affected overall as expected by the visual, oculomotor and sensorimotor impairments frequently associated with MALNS [2], these abilities appear consistent with their general intellectual functioning.

The transformation of raw scores into scaled scores avoiding approximation to the lowest values allowed for the identification of strengths and weaknesses. Without this methodological choice, the presence of ID across participants would have yielded a floor effect in almost all subtests, limiting the delineation of a specific profile. In this light, the use of normative standardisation tables for chronological age as usually performed in clinical settings may not provide an accurate description of the cognitive functioning of individuals with MALNS [27]. Furthermore, the presence of speech impairments hinders the full administration of many commonly used batteries for assessing ID, increasing the risk of misclassification. While the use of nonverbal tests represents a feasible alternative for evaluating IQ [10], it provides only a partial description of cognitive functioning. In this context, clinician-informed measures, standardised parent-reports or even self-reports tailored to the needs of people with ID may offer a more practical and informative choice for evaluating cognitive and social functioning in individuals with MALNS [28,29].

Some limitations should be considered when interpreting the findings of the present study. First, although the sample size aligns with previous studies on this ultra-rare syndrome [11], the small cohort warrants caution in generalising the results. Nonparametric tests were employed to mitigate the impact of the small sample size, allowing the identification of specific strengths and weaknesses in the neuropsychological profile. Nonetheless, the findings presented here should be considered as exploratory and confirmed in larger samples. While this study employed a cross-sectional design, longitudinal data would provide valuable insights into the developmental trajectories of cognitive and social functioning in MALNS, from childhood to adulthood [1]. The use of a single, co-normed battery allowed the overcoming of issues of previous studies [10]; however, direct comparisons with other clinical populations could enrich the understanding of social–cognitive impairments in MALNS [30]. Moreover, speech and cognitive impairments prevented the administration of the full NEPSY-II battery, precluding the assessment of other skills such as inhibition and auditory attention. Lastly, although our findings on social perception suggest differences from ASD, and all participants underwent a psychopathological assessment in a previous study [12], we eventually did not perform a formal clinical assessment of ASD because this goal was outside the scope of this study. Social perception skills should be evaluated in combination with clinical measures of autism symptomatology.

Despite these limitations, the results of this study provide new evidence of specific cognitive and social features in MALNS, contributing to a more detailed understanding of the neuropsychological profile associated with this ultra-rare syndrome. Tailoring interventions to these distinctive features may promote better outcomes in both educational and daily life contexts. The observed facilitation for visual over verbal tasks highlights the importance of designing interventions that leverage picture-based stimuli to support comprehension and communication, especially for individuals with significant speech impairments. In this light, augmentative and alternative communication approaches, such as the use of visual schedules, communication boards and digital devices, may be particularly beneficial in fostering expressive and receptive communication skills [31]. Similarly, behavioural interventions that incorporate visual and concrete stimuli, such as token economy systems and visual timers, appear well-suited to address challenges in emotional regulation and to facilitate participation in daily activities. These tools can provide clear, immediate feedback and structure, which are crucial for reducing anxiety and increasing predictability in interactions [32]. Such interventions may be further adapted to meet the individual needs of children and adolescents with MALNS by using meaningful and familiar pictures or objects according to their preferences and strengths. Moreover, the identified strength in memory for facial stimuli suggests that individuals with MALNS may be particularly sensitive to social reinforcements, including positive facial expressions, physical touch and gestures [33]. These social reinforcements can be systematically integrated into behavioural interventions, not only to increase engagement and learning but also to foster social connections and improve adaptive social skills in educational and daily life settings [34].

## Figures and Tables

**Table 1:** Main clinical features of the six recruited individuals with MALNS.

Id	1	2	3	4	5	6
**Gender**	M	F	M	M	F	M
**Age** (years)	7.6	18.9	17.6	8.9	10.1	14.3
**Genetic feature**	Microdeletions	Intragenic variants	Microdeletions	Intragenic variants	Intragenic variants	Intragenic variants
**Intellectual disability**	Moderate	Severe	Severe	Moderate	Severe	Mild
**Visual problems**	X	X	X	X		X
**EEG anomalies/seizures**	X			X		
**Speech impairments**	X		X		X	
**Chiari I malformation**				X		X
**Nonverbal IQ (Alfieri et al., 2022 [10]** **)**	65	47	45	52	54	62
**Adaptive Behaviour Composite** **—** **Vineland II (Alfieri et al., 2022 [10]** **)**	39	20	20	42	23	56
**Neurodevelopmental and behavioural disorders (Alfieri et al., 2023 [12]** **)**	Separation anxiety	Anxiety	Anxiety	Irritability	ADHD	ADHD

**Table 2:** Results of the neuropsychological assessment. Scores are reported as median (range).

Subtest	Main Assessed Ability/Behaviour	Scaled Score	Participants Showing Weaknesses
Visual attention	Visual, selective attention	2.2 (−5.2–5.8)	3 (#2, 3, 5)
Comprehension of instructions	Receptive language	−7.0 (−30.0–0.1)	6
Memory for faces	Encoding and retrieval of facial stimuli	2.5 (−1.5–13.6)	3 (#3, 4, 5)
Memory for designs	Visual–spatial memory	−7.1 (−18.8–2.5)	6
Manual motor sequences	Encoding and retrieval of rhythmic motor programmes	−5.9 (−26.3–−0.8)	6
Theory of mind—Verbal part (A)	Understanding mental functions (e.g., belief, pretending)	−9.5 (−22.2–0.8)	6
Theory of mind—Contextual part (B)	Understanding others’ mental states according to context	0.5 (−6.8–−7.2)	4 (#2, 3, 4, 5)
Affect recognition	Facial affect recognition	−2.8 (−3.8–−0.3)	6
Block construction	Visuospatial construction skills	−2.5 (−3.0–0.6)	6
Geometric puzzles	Mental rotation of abstract stimuli	−4.2 (−16.2–1.1)	6

**Table 3:** Results of the Wilcoxon matched-pairs tests between the NEPSY-II subtests. Significant *p* values (<0.05) are reported in bold.

Subtest		CI	MF	MD	MMS	ToMA	ToMB	AR	BC	GP
**VA**	*Z*	2.20	0.74	2.20	2.20	2.20	0.52	1.78	1.36	1.99
	*p*	**0.028**	0.463	**0.028**	**0.028**	**0.028**	0.600	0.075	0.173	**0.046**
**CI**	*Z*		2.20	0.73	0.52	0.10	2.20	1.99	2.20	1.57
	*p*		**0.028**	0.463	0.600	0.912	**0.028**	**0.046**	**0.028**	0.116
**MF**	*Z*			2.20	2.20	2.20	1.15	2.20	2.20	1.99
	*p*			**0.028**	**0.028**	**0.028**	0.249	**0.028**	**0.028**	**0.046**
**MD**	*Z*				0.73	0.73	1.99	1.15	1.57	0.73
	*p*				0.463	0.463	**0.046**	0.249	0.116	0.463
**MMS**	*Z*					0.31	2.20	1.57	1.57	0.94
	*p*					0.753	**0.028**	0.116	0.116	0.345
**ToMA**	*Z*						2.20	1.99	1.99	1.99
	*p*						**0.028**	**0.046**	**0.046**	**0.046**
**ToMB**	*Z*							1.57	0.94	1.99
	*p*							0.116	0.345	**0.046**
**AR**	*Z*								1.15	1.15
	*p*								0.249	0.249
**BC**	*Z*									1.57
	*p*									0.116

Legend: VA = Visual Attention; CI = Comprehension of Instructions; MF = Memory for Faces; MD = Memory for Designs; MMS = Manual Motor Sequences; ToMA = Verbal part of Theory of Mind; ToMB = Contextual part of Theory of Mind; AR = Affect Recognition; BC = Block Construction; GP = Geometric Puzzles.

## Data Availability

The raw data supporting the conclusions of this article are publicly available at this link: https://osf.io/93428/.

## Abbreviations

The following abbreviations are used in this manuscript:

MALNS	Malan syndrome
ID	Intellectual Disability
ADHD	Attention Deficit and Hyperactivity Disorder
ASD	Autism Spectrum Disorder
ToM	Theory of Mind
IQ	Intelligent Quotient
ASSI	Associazione Sindrome di Sotos Italia
VA	Visual Attention
CI	Comprehension of Instructions
MF	Memory for Faces
MD	Memory for Designs
MMS	Manual Motor Sequences
AR	Affect Recognition
BC	Block Construction
GP	Geometric Puzzles
SD	Standard Deviation
